# Respectful midwifery care during the COVID-19 pandemic

**DOI:** 10.18332/ejm/120070

**Published:** 2020-04-08

**Authors:** Victoria G. Vivilaki, Eleni Asimaki

**Affiliations:** 1 Midwifery Department, University of West Attica, Athens, Greece

**Keywords:** COVID-19, pregnancy, breastfeeding, stigma, philosophy, medicalization

The COVID-19 pandemic is affecting all areas of perinatal care, and midwives are facing enormous challenges. The International Council of Midwives (ICM) recently expressed concerns regarding the violation of the human rights of women, neonates and midwives, with increasing cases of caesarean sections, not initiating breastfeeding and isolating mothers from their birth partners and newborns^1^.

Misconceptions among healthcare professionals lead to unnecessary interventions in childbirth^2^ and possible institutional stigma^2^. Even though COVID-19 per se is not a contraindication for a vaginal birth, women with COVID-19 give birth by caesarean section^3^ possibly due to different perceptions and fear of complications and transmission. Also, women with COVID-19 might tend to get less involved in decision making in childbirth, while their concerns and possible fear of birth might make them request a caesarean section themselves. The healthcare providers’ fear of the unknown not only fuels stigma but also emphasises the concept of risk management and categorisation, as an attempt to minimise the uncertainty and shape a more predictable future^4^. Therefore, within this context, health professionals resort to medicalised deliveries, based on the belief that in this way they have more control over the birth process^5^. In fact, any intervention in childbirth – in terms of defensive medicine – generates a cascade of interventions, interrupts the physiological labour process and creates a higher risk for maternal and neonatal adverse outcomes^6^. Within today’s blame-culture, the mother-to-be also feels accountable for her baby’s health and is more willing to undergo further monitoring and interventions^7^. Moreover, it has been advocated that maternal choice is influenced by sociocultural factors and the obstetric discourse that is dominant at a specific time^8^.

All the above could, at least partly, explain the way pregnant women are managed (with suspected/diagnosed COVID-19 infection or even healthy during the current crisis). We should recognise that pregnant and labouring women form a vulnerable, but not homogenous, group with fundamental human rights to dignity and respectful, individualised midwifery care, which safeguards both the physical and mental health of the mother and baby dyad. Even if there is a need for further monitoring and interventions, it is essential to provide woman-centred care, establish good communication with mothers and offer emotional support and stress management^9^.

In these challenging times, pregnant women and mothers should not feel less safe and discouraged from making decisions for themselves and their babies. Dissemination of evidence-based information, adherence to the official clinical guidelines and recommendations, education and skills training of healthcare providers should all be promoted at a professional, organisational and governmental level. Especially under these circumstances, the role of the midwife is more recognised as an advocate of natural birth for women^10^, and a key professional in understanding healthcare of women with COVID-19 and all its complexities, in order to provide a theoretical evaluation of the ‘medicalised terminology’ and the underpinning philosophy.
